# Characterisation of Growth Plate Dynamics in Murine Models of Osteoarthritis

**DOI:** 10.3389/fendo.2021.734988

**Published:** 2021-10-20

**Authors:** Hasmik J. Samvelyan, Kamel Madi, Anna E. Törnqvist, Behzad Javaheri, Katherine A. Staines

**Affiliations:** ^1^ School of Pharmacy and Biomolecular Sciences, University of Brighton, Brighton, United Kingdom; ^2^ Centre for Stress and Age-Related Disease, University of Brighton, Brighton, United Kingdom; ^3^ The Faculty of Health, Education, Medicine and Social Care, School of Medicine, Anglia Ruskin University, Chelmsford, United Kingdom; ^4^ 3Dmagination Ltd, Harwell Campus, Didcot, United Kingdom; ^5^ Rheumatology and Bone Diseases Unit, Centre for Genomic and Experimental Medicine, Medical Research Council (MRC) Institute of Genetics and Molecular Medicine, University of Edinburgh, Edinburgh, United Kingdom; ^6^ Centre for Bone and Arthritis Research, University of Gothenburg, Gothenburg, Sweden; ^7^ School of Mathematics, Computer Science and Engineering, City University of London, London, United Kingdom

**Keywords:** osteoarthritis, cartilage, growth, chondrocyte, murine model

## Abstract

The purpose of this study was to investigate growth plate dynamics in surgical and loading murine models of osteoarthritis, to understand whether abnormalities in these dynamics are associated with osteoarthritis development. 8-week-old C57BL/6 male mice underwent destabilisation of medial meniscus (DMM) (*n =* 8) surgery in right knee joints. Contralateral left knee joints had no intervention (controls). In 16-week-old C57BL/6 male mice (*n* = 6), osteoarthritis was induced using non-invasive mechanical loading of right knee joints with peak force of 11N. Non-loaded left knee joints were internal controls. Chondrocyte transiency in tibial articular cartilage and growth plate was confirmed by histology and immunohistochemistry. Tibial subchondral bone parameters were measured using microCT and correlated to 3-dimensional (3D) growth plate bridging analysis. Higher expression of chondrocyte hypertrophy markers; Col10a1 and MMP13 were observed in tibial articular cartilage chondrocytes of DMM and loaded mice. In tibial growth plate, Col10a1 and MMP13 expressions were widely expressed in a significantly enlarged zone of proliferative and hypertrophic chondrocytes in DMM (*p*=0.002 and *p*<0.0001, respectively) and loaded (both *p*<0.0001) tibiae of mice compared to their controls. 3D quantification revealed enriched growth plate bridging and higher bridge densities in medial compared to lateral tibiae of DMM and loaded knee joints of the mice. Growth plate dynamics were associated with increased subchondral bone volume fraction (BV/TV; %) in medial tibiae of DMM and loaded knee joints and epiphyseal trabecular bone volume fraction in medial tibiae of loaded knee joints. The results confirm articular cartilage chondrocyte transiency in a surgical and loaded murine models of osteoarthritis. Herein, we reveal spatial variation of growth plate bridging in surgical and loaded osteoarthritis models and how these may contribute to anatomical variation in vulnerability of osteoarthritis development.

## Introduction

Osteoarthritis (OA) is a chronic musculoskeletal disease and a leading cause of disability and major healthcare costs in the world. It is estimated that worldwide 10% of men and 18% of women aged over 60 years have symptomatic OA (1) and, therefore, the predicted increase in the ageing population and longevity will result in a greater occurrence of the disease. OA is a complex disease in which the pathogenesis, cellular and molecular mechanisms of initiation and progression are not completely understood. It is characterised by progressive loss of articular cartilage (AC), formation of osteophytes, subchondral bone (SCB) sclerosis, synovial proliferation, inflammation and lax tendons. These can ultimately lead to a loss of joint function, pain, reduced mobility and disability (2). Despite the significant healthcare and economic burden there are few non-invasive therapies available to patients. Therefore, understanding the pathogenesis of OA and defining the molecular mechanisms underpinning AC degeneration can lead to the development of successful targeted and effective disease-modifying treatments.

Primary OA is described as naturally occurring OA affecting one joint (localised) or three or more joints (generalised), while secondary OA is associated with various causes and risk factors leading to the disease including trauma, obesity, diabetes, metabolic bone and congenital disorders (3). AC degeneration is one of the main hallmarks of OA and previous research has largely sought to identify mechanisms underpinning its deterioration. Fully developed, uncalcified AC is populated by a single resident cell chondrocytes, which maintain a stable phenotype characterised by small cell size and expression of tenascin-C (4). The inherent stability of AC chondrocytes ensures that dynamic events are restricted to assure lifelong articular integrity and healthy joint function. In contrast, epiphyseal growth plate (GP) chondrocytes have a transient phenotype to ensure long bone development (endochondral ossification) and growth. GP dynamics are defined by the GP chondrocytes which undergo a differentiation sequence of proliferation, maturation and hypertrophy. The final stage of chondrocyte hypertrophy enables mineralisation of the cartilage extracellular matrix, vascular invasion and subsequent replacement of the mineralised cartilage anlagen with bone (5). With sexual maturation the human GP undergoes progressive narrowing as GP bony bridges form and span its width, establishing continuity between the cancellous bone of the epiphysis and metaphysis. These GP bridging events ultimately lead to complete GP closure and cessation of human growth (6). In mice, longitudinal bone growth does not cease at sexual maturity instead it slows dramatically at puberty, but the GPs do not completely fuse and disappear (7).

We have previously shown that in the STR/Ort mouse, a naturally occurring OA murine model, AC chondrocytes transform from their inherently stable phenotype to a transient one, characteristic of the chondrocytes in the GP. This was confirmed by immunolabelling for chondrocyte hypertrophy markers; type X collagen (Col10a1) and matrix metalloproteinase 13 (MMP13) (8). Further, we revealed accelerated longitudinal bone growth, aberrant expression of GP markers (Col10a1 and MMP13) and increased GP chondrocyte maturation in these mice. Consistent with this, using a novel synchrotron computed tomography method we revealed enriched GP bone bridging in STR/Ort mouse tibiae indicative of advanced GP closure which may underpin OA (8, 9). However, recently it has been shown that the surgical induction of OA resulted in disruption of GP organisation in wild type and Ellis-van-Creveld syndrome protein (Evc) knockout mice (model of GP disorganisation due to disrupted hedgehog signalling), but did not exacerbate AC damage (10). In this study, the authors reported an altered columnar structure of chondrocytes within the GP, with clusters of chondrocytes in the DMM model, which was exacerbated in the DMM-Evc animals. This was associated with disrupted Col10a1 and MMP13 expressions in the GP of these animals. However, unlike in the STR/Ort mouse, disrupted GP structure was not associated with increased AC damage (8, 10). Thus, interlinks between the differing chondrocyte phenotypes in the AC and the GP, and the precise contribution that the GP and its fusion mechanisms may play in underpinning OA development is not yet fully understood.

Therefore, the aim of this study was to build on previous research into the significance of the GP in OA, through investigating whether non-surgically (mechanical loading) induced OA in C57BL/6 mice is linked to altered GP dynamics, and specifically GP bridging events which may further drive OA development. We also aimed to further explore associations between surgically (destabilisation the medial meniscus [DMM]) induced OA and alterations in the GP. Indeed, understanding this will inform strategies for maintaining musculoskeletal health in ageing by adding further evidence towards whether there is an association between longitudinal bone growth and OA.

## Materials and Methods

### Animals

Male C57BL/6 wild type mice at 7 weeks of age (young adult) were obtained from Charles River Laboratories Inc. (Margate, UK). The mice were acclimatised to their surroundings for seven days. All mice were allowed free access to water and maintenance diet ad libitum (Special Diet Services, Witham, UK) in a 12-hour light/dark cycle at a room temperature of 21 ± 2°C and relative humidity of 55 ± 10%. The animal study was approved by the Roslin Institute Animal Users and Research Ethics Committees, and the animals were maintained in accordance with UK Home Office guidelines for the care and use of laboratory animals. Animal studies were conducted in line with the ARRIVE guidelines.

### The Destabilisation of the Medial Meniscus (DMM)

8-week-old wild type C57/BL6 male mice underwent DMM surgeries to induce OA-like changes in the right knee joints under isoflurane-induced anaesthesia (*n* = 8/group). We chose not to performed SHAM (placebo) surgery on the left contralateral knee of the animals based upon animal welfare grounds in keeping with the 3Rs, since it was previously shown that there is no difference in OA scores between SHAM-operated and non-operated knee joints (11, 12). Animals were monitored for unexpected adverse effects to reduce suffering and distress. Following transection of the medial meniscotibial ligament (MMTL) to destabilise the medial meniscus, the skin was closed and anaesthesia reversed (11). Eight weeks later, 16-week-old mice were culled by exsanguination and confirmation of death by cervical dislocation. The knee joints of all the mice were dissected, fixed in 4% paraformaldehyde for 24 hours at 4°C, and then stored in 70% ethanol.

### *In Vivo* Loading of the Knee Joint

The right knee joints of 16-week-old wild type C57BL/6 male mice (*n* = 6) were subjected to non-invasive, dynamic axial mechanical loading under the isoflurane-induced anaesthesia (liquid isoflurane was vaporised to a concentration of 4% and maintained at a concentration of 2% with oxygen) for 7 min/day, 3 alternate days a week for 2 weeks according to the protocols described in the previous studies (13, 14). The left knee joints were non-loaded internal controls in these animals. Briefly, using a servo-electric materials testing machine (Electroforce 3100, Bose, UK), axial compressive loads were applied through the right knee joint *via* customised concave cups which held the knee and ankle joints flexed and the tibiae vertically.

The tibia was held in place by continuous static preload of 0.5N onto which dynamic loads were superimposed in a series of 40 trapezoidal shaped waveform cycles with steep up and down ramps and a peak force of 11N for 0.05 seconds (0.025 seconds rise and fall time; 9.9 seconds baseline hold time between periods of peak loading). The right and left knees were dissected 3 days after the final loading episode. Mice were culled by exsanguination and confirmation of death by cervical dislocation. Knee joints were fixed in 4% paraformaldehyde for 24 hours at 4°C before being stored in 70% ethanol.

### Micro-Computed (MicroCT) Tomography and 3-Dimensional (3D) Bridging Analysis

Scans were performed with an 1172 X-Ray microtomograph (Bruker MicroCT, Kontich, Belgium) to evaluate the SCB and GP bridging. High-resolution scans with an isotropic voxel size of 5 µm were acquired (50 kV, 200 µA, 0.5 mm aluminium filter, 0.6° rotation angle). The projection images were reconstructed and binarised with a threshold of 0 to 0.16, ring artefact reduction was set at 10 and beam hardening correction at 0% using the SkyScan NRecon software package (v1.6.9.4, Bruker MicroCT). The images then were realigned vertically using DataViewer software (v1.5.1.2 64-bit, Bruker MicroCT) to ensure similar orientation for analysis. Hand-drawn regions of interests (ROI) of the SCB and epiphyseal trabecular bone for each tibial lateral and medial compartments were selected (15). The structural parameters of tibial SCB plate and epiphyseal trabecular bone were calculated using 3D algorithms of SkyScan CTAn software (Bruker MicroCT) including SCB (SCB BV/TV; %) and trabecular bone volume fraction (Tb. BV/TV; %) and correlated to GP bridging analysis using a 3D synchrotron-computed tomography quantification method as previously described (16). Briefly, microCT scans of the tibiae were segmented using a region-growing algorithm within the Avizo^®^ (V8.0, VSG, Burlington, VT, USA) software. The central points of each bony bridges were identified and projected on the tibial joint surface. The distribution of the areal number density of bridges (N, the number of bridges per 256 µm × 256 µm window; *d* = *m*/*V*) is then calculated and superimposed on the tibial joint surface (each bridge has a colour that represents the areal number density at the bridge location). The SCB plate and epiphyseal trabecular bone thickness (Th; mm) was determined and colour-coded thickness images were generated using the Avizo^®^ software.

### Histological Analysis

The left and right knee joints of all the mice were decalcified in 10% ethylenediaminetetraacetic acid (EDTA) solution for 4 weeks at 4°C, wax-embedded at Leica EG1160 Tissue Embedding Station and 6 μm coronal sections cut using Leica RM2135 manual microtome.

### Immunohistochemistry

Immunohistochemical analysis of established chondrocyte transiency markers in tibial AC and GP was performed on 6 μm coronal sections from the middle region of the knee joint, using anti-matrix metalloproteinase 13 (anti–MMP13) (1:200 dilution; Abcam) or anti-collagen type X (anti-Col10a1) (1:100 dilution; Abcam) antibodies. As a control, an equal concentration of rabbit IgG was used. For immunohistochemical localisation of MMP13 and Col10a1, sections were dewaxed in xylene and rehydrated. Sections were incubated at 37°C for 30 min in 1mg/ml trypsin for antigen demasking. Endogenous peroxidases were blocked by treatment with 0.3% H_2_O_2_ in methanol (Sigma) for 30 min at room temperature. The Vectastain ABC universal detection kit (Vector Laboratories, Peterborough, UK) was used to detect the biotinylated secondary antibody (Anti-Mouse IgG Reagent) after incubation for 30 min at room temperature according to the manufacturer’s instructions. Diaminobenzidine (DAB) solution used to detect the location of antigens. The sections were finally dehydrated, counterstained with haematoxylin and mounted in DePeX. All sections to be compared were immunolabelled at the same time to standardise conditions and minimise any differences in antibody incubation times. For each group, immunolabelling was performed on four individual animals per experimental group and representative images taken using a light microscope.

### Growth Plate (GP) Zone Analysis

The width of the GP proliferating and hypertrophic zones, as well as the total GP width, were measured at 10 different points along the length of the GP based on established cell morphology (17) in 6μm coronal sections from the middle region of the knee joint in similar location of four individual animals per experimental group, using a light microscope and ImageJ (NIH, Bethesda, MD, USA) software.

### Statistical Analysis

All analyses were performed with GraphPad Prism software 6.0f version (GraphPad Inc, La Jolla, CA, USA) using a two-sided 0.05 level of significance. All analyses were conducted blindly to minimise the effects of subjective bias. The results were presented as the mean ± standard error of the mean (SEM). The Normal distribution of data was assessed using the Shapiro-Wilk normality test. For comparing two groups (experimental with control, or medial with the lateral compartment of tibiae), two-tail Student’s *t*-test (paired or unpaired) was used. For comparing more than two groups, two-way ANOVA (analysis of variance) was used with Tukey post-hoc test.

## Results

### Transient Chondrocyte Behaviour in the Tibial AC of DMM and Loaded C57BL/6 Young Adult Male Mice

**Figure 1:**
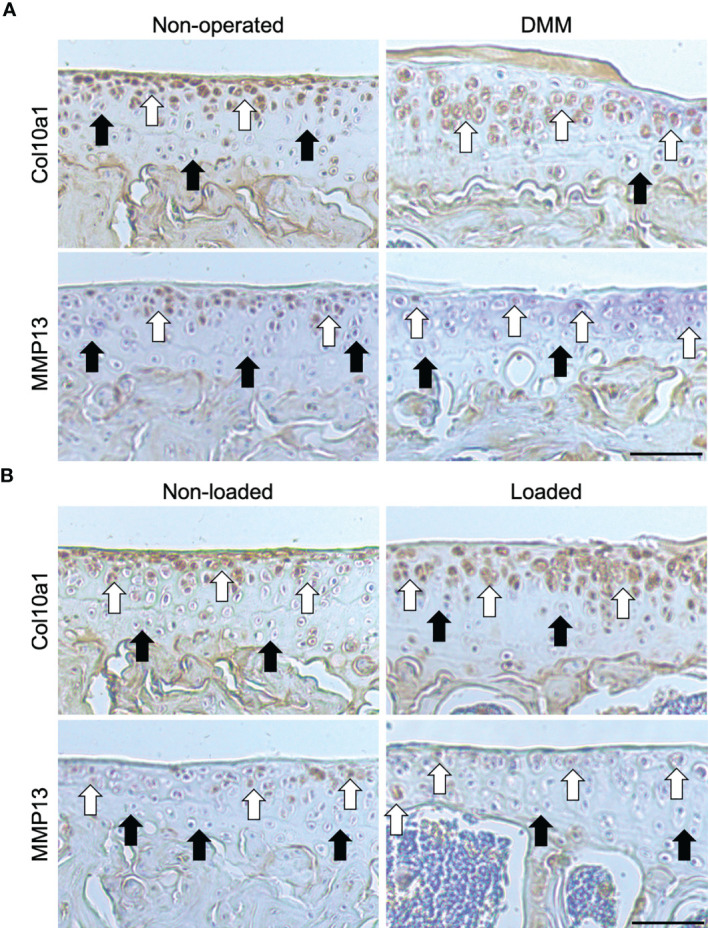
Immunohistochemical labelling in the tibial articular cartilage of mouse knee joints. Immunohistochemical labelling for type X collagen (Col10a1) and matrix metalloproteinase (MMP13) in the 6μm coronal sections of articular cartilage of non-operated and DMM **(A)**, or non-loaded and mechanically loaded **(B)** middle regions of knee joints of C57BL/6 male mice. White arrows indicate examples of positive labelling, and black arrows indicate a lack of labelling, in the tibiae. Images are representative of results in 4 individual mice. Scale bar = 50μm.

We first sought to confirm whether loss of AC in C57BL/6 mice with surgically and loading induced OA was associated with the expression of markers of transient chondrocyte phenotype. In accordance with previous studies, immunohistochemistry analysis showed higher expression levels of well-established chondrocyte hypertrophy markers; Col10a1 and MMP13, observed in tibial AC of C57BL/6 mice that have undergone DMM surgery or mechanical loading compared with non-operated and non-loaded control left tibiae, respectively (**Figures 1A, B**). The expression pattern of Col10a1 was largely restricted to hypertrophic chondrocytes in the uncalcified zone of the AC of unaffected condyles of non-operated (**Figure 1A**) and non-loaded (**Figure 1B**) mouse left joints (18). Whereas, immunolabeling of Col10a1 was more widespread throughout the chondrocytes of both the uncalcified and calcified cartilage and the extracellular matrix (ECM) of the AC in affected right joints of DMM (**Figure 1A**) and loaded (**Figure 1B**) mice. Similarly, immunohistochemistry analysis showed positive MMP13 labelling in both superficial and deep articular chondrocytes in the right joints of DMM and loaded C57BL/6 male mice compared to the control knee joints (**Figures 1A, B**). These findings confirm an aberrant deployment of transient chondrocytes in uncalcified AC.

### Dysfunctional GP Dynamics in DMM and Loaded Tibiae of C57BL/6 Young Adult Male Mice

**Figure 2:**
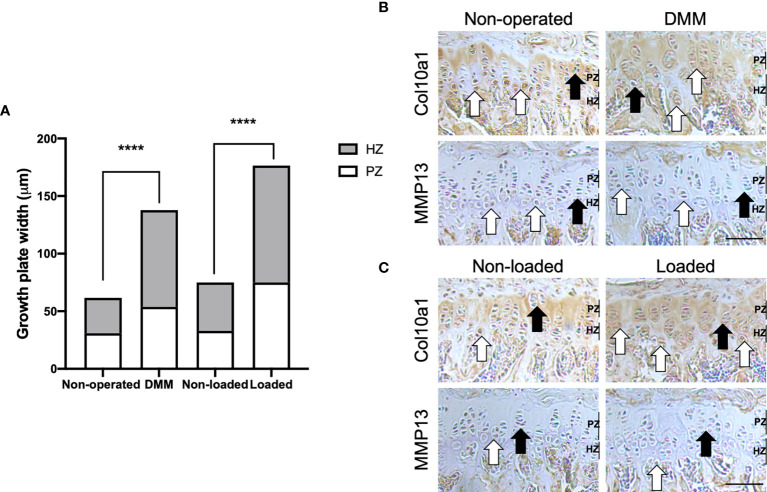
Growth plate dynamics in the tibial growth plate of mouse knee joints. Growth plate zone width of non-operated and DMM, or non-loaded and loaded knee joints of C57BL/6 male mice **(A)**. Ten measurements per section were obtained along the length of the tibial growth plate in the middle region of the knee joint (n=4 mice for each experimental group). Immunohistochemical labelling for type X collagen (Col10a1) and matrix metalloproteinase (MMP13) in the 6μm coronal sections of growth plate of DMM and non-operated **(B)**, or mechanically loaded and non-loaded **(C)** middle regions of knee joints of C57BL/6 male mice. White arrows indicate examples of positive labelling, and black arrows indicate a lack of labelling, in the tibiae. Images are representative of results in 4 individual mice. Scale bar = 100μm. PZ, proliferative zone; HZ, hypertrophic zone ****p < 0.0001.

To determine the GP phenotype in these OA models, we first completed histological assessment of GP zones. Our analysis revealed significantly enlarged proliferative and hypertrophic zones of chondrocytes in both DMM (*p*=0.002 and *p*<0.0001, respectively) and loaded (both *p*<0.0001) tibiae of C57BL/6 mice compared to their controls, and significantly increased total GP width (both *p*<0.0001) (**Figure 2A**). This is suggestive of aberrant GP dynamics in these OA models.

We next determined the expression of known chondrocyte transiency markers in the GPs. In the tibial GP of mice with surgically and loading induced OA, Col10a1 expression was more greatly and widely dispersed throughout the zones of proliferative and hypertrophic chondrocytes compared with their controls (**Figures 2B, C**). Indeed, immunolabeling for Col10a1 revealed the expected localisation in the GP of non-operated and non-loaded mouse tibiae, limited primarily to the hypertrophic zone and underlying adjacent metaphyseal bone (**Figures 2B, C**). Differing distribution of a GP zone marker was also evident for MMP13 in the GP of DMM and loaded C57BL/6 mouse tibiae compared to their controls (**Figures 2B, C**). Together, the results may indicate associations between dysfunctional GP morphology and marker expression, and disease development in these murine models of OA.

### Associations Between GP Bridging in DMM and Loaded Tibiae of C57BL/6 Young Adult Male Mice and OA Development

**Figure 3:**
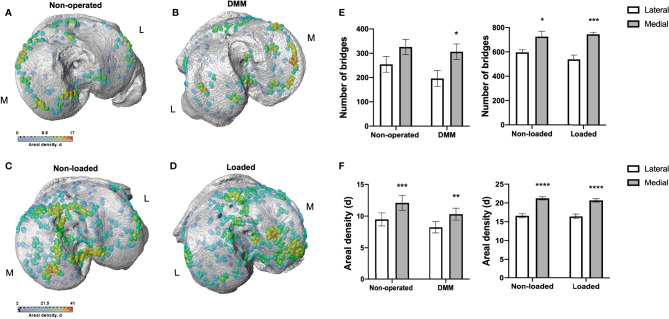
Bridge location and bridge areal densities across the tibial growth plate of mice. Location and areal densities of bridges across the growth plate projected on the medial (M) and lateral (L) tibial joint surface in non-operated **(A)**, DMM **(B)**, non-loaded **(C)** and loaded **(D)** tibiae of mice at 16 and 18 weeks of age, number of bridges in lateral and medial tibiae of non-operated compared to DMM and non-loaded compared to loaded tibiae of mice **(E)**, Areal density (d) of bridges in medial compared to lateral tibiae, defined as the number of bridges per 256 mm x 256 mm window of non-operated and DMM or non-loaded and loaded knee joints **(F)**. Bars represent mean ± SEM. Group sizes were *n* = 8 for non-operated and DMM-operated mice and *n* = 6 for non-loaded and loaded mice. * indicates differences between lateral and medial tibiae *p < 0.05 **p < 0.01 ***p < 0.001 ****p < 0.0001.

To further correlate aberrant longitudinal GP dynamics, GP bridging and OA development in these models, we used our newly developed 3D method to quantify GP bone bridges across the tibial epiphysis of DMM and loaded mice for the first time (**Figure 3**). 3D quantification revealed a significantly higher number of GP bridges in medial compared to lateral tibiae that underwent DMM surgeries (196 ± 33 *versus* 306 ± 32; *p*=0.03) (**Figures 3B, E**). This significant difference was not observed in non-operated tibiae (254 ± 32 *versus* 326 ± 32; *p*=0.14) (**Figures 3A, E**). Similarly, significantly enriched GP bridging was evident in the medial compartment of loaded tibiae in comparison to the lateral compartment (539 ± 36 *versus* 745 ± 18; *p*=0.0009) (**Figures 3D, E**), and in those of non-loaded tibiae, although less pronounced than in the loaded right knee joints (596 ± 22 *versus* 726 ± 44; *p*=0.04) (**Figures 3C, E**). However, no significant differences in GP bridge numbers and densities were observed between interventions (DMM *versus* non-operated and loaded *versus* non-loaded) at this time point in either the medial or lateral compartment.

These results were consistent with the areal bridge density analysis. The mean areal bridge densities were significantly greater in medial compared to the lateral compartment of DMM (8.2 ± 0.9 *versus* 10.3 ± 0.9; *p*=0.01) and loaded tibiae (16.3 ± 0.6 *versus* 20.6 ± 0.5; *p*<0.0001) (**Figure 3F**). However, no significant differences in the mean areal bridge densities were observed between interventions (DMM *versus* non-operated, and loaded *versus* non-loaded).

Anatomical variations were observed however in all samples, with focal high density clusters forming in the anterior region of the medial loaded tibiae *versus* those more in the central and posterior of non-loaded tibiae (**Figures 3C, D**). Similarly, in the DMM tibiae, GP bridges were more widespread across the tibiae than in non-operated which were predominantly observed around the periphery (**Figures 3A, B**). Together, these results suggest that murine OA models exhibit spatial variations in GP bridging in comparison to controls.

### MicroCT Analysis and 3D Visualisation of SCB Plate and Epiphyseal Trabecular Bone

**Figure 4:**
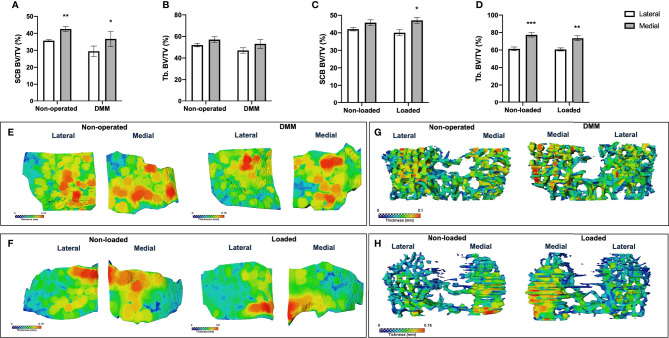
MicroCT analysis of epiphyseal region of medial and lateral tibiae of mice. MicroCT analysis of the epiphyseal region of the lateral and medial tibiae in non-operated controls and DMM-operated knee joints subchondral bone volume fraction (SCB BV/TV) **(A)** and epiphyseal trabecular bone volume fraction (Tb. BV/TV) **(B)**. MicroCT analysis of the epiphyseal region of the medial and lateral tibiae in non-loaded controls and loaded knee joints subchondral bone volume fraction (SCB BV/TV) **(C)** and epiphyseal trabecular bone volume fraction (Tb. BV/TV) **(D)**. Representative colour coded images of lateral and medial subchondral bone plate thickness of non-operated and DMM-operated tibiae of mice **(E)**. Representative colour coded images of lateral and medial subchondral bone plate thickness of non-loaded and loaded tibiae of mice **(F)**. Representative colour coded images of epiphyseal trabecular bone thickness of non-operated and DMM-operated **(G)**, or non-loaded and loaded tibiae of mice **(H)**. Bars represent mean ± SEM. Group sizes were *n* = 8 for non-operated and DMM-operated mice and *n* = 6 for non-loaded and loaded mice. * indicates differences between lateral and medial tibiae *p < 0.05 **p < 0.01 ***p < 0.001.

To establish whether spatial variations in GP bridging are associated with the SCB plate and epiphyseal trabecular bone abnormalities after the DMM surgery and mechanical loading in our mice, we performed microCT analysis and determined local thickness of SCB plate and epiphyseal trabecular bone. The SCB plate volume fraction (SCB BV/TV) was significantly higher in medial compared to lateral compartment of DMM and non-operated tibiae (DMM: SCB BV/TV 29.4 ± 3.1% *versus* 36.7 ± 4.5%, *p*=0.03, non-operated: SCB BV/TV 35.7 ± 0.7% *versus* 42.6 ± 1.5%, *p*=0.004) (**Figure 4A**). No significant differences were observed between DMM and non-operated tibia (**Figure 4A**), or in the epiphyseal trabecular bone volume fraction (Tb. BV/TV; **Figure 4B**). Conversely, in the loaded and non-loaded tibiae, epiphyseal trabecular bone volume fraction (Tb. BV/TV) was significantly increased in the medial compared to lateral compartment (loaded: Tb. BV/TV 60.7 ± 1.8% *versus* 73.4 ± 2.8%, *p*=0.005, non-loaded: Tb. BV/TV 61.4 ± 2.2% *versus* 77.4 ± 2.5%, *p*=0.0005). No significant differences were observed between interventions (**Figure 4D**). The SCB plate volume fraction (SCB BV/TV) was significantly higher in medial compared to lateral compartment of loaded tibiae (40.1 ± 1.7% *versus* 47.1 ± 1.6%, *p*=0.02), but not that of non-loaded tibiae (42.1 ± 1.1% *versus* 45.8 ± 1.6%, *p*=0.34) or between interventions (**Figure 4C**).

Colour-coded SCB plate thickness analysis revealed anatomical variation in SCB plate thickness between DMM and non-operated, and loaded and non-loaded plates (**Figures 4E, F**). Differences in the non-loaded and loaded SCB plates were particularly apparent (**Figure 4F**), and correlated with the clusters of higher density of GP bridges previously observed (**Figures 3C, D**). Epiphyseal trabecular bone thickness was not significantly altered in response to either invasive or non-invasive intervention (data not shown; **Figures 4G, H**).

## Discussion

Together, the data presented herein reveal altered GP dynamics in both surgical (DMM) and non-invasive loading murine *in vivo* models of OA. Our data confirm previous studies showing changes in AC of the knee joints of these mice consistent with the aberrant deployment of chondrocyte transiency, and reveal disrupted GP morphology, increased GP chondrocyte differentiation indicated by widespread expression of chondrocyte hypertrophy markers and increased GP zone widths in these models. Moreover, we have utilised a recently developed 3D model to discover for the first time anatomical variations in GP bone bridging, indicative of GP fusion, which are spatially correlated with increase in SCB thickness. These data reveal that altered GP dynamics and spatial differences in GP bridging may contribute to an anatomical variation in vulnerability of OA development in surgical and loaded murine models of OA which with findings described above may indicate an endochondral defect in AC and GP cartilage in these mouse models of OA. These findings build upon our previous work in the STR/ort OA murine model, and on work by Lameudra et al., in the Evc murine model (8, 10), and further highlight the contribution that the GP may play in OA development.

The STR/ort mouse is predisposed to developing spontaneous idiopathic OA whilst its nearest available parental strain, the CBA mouse, has a very low susceptibility which makes them effective controls for the studies (19). We have previously shown that aberrant deployment of transient chondrocyte behaviour, consistent with re-initiation of endochondral processes, occurs in uncalcified AC of STR/ort mouse knee joints compared to CBA controls (8). Here we extend these studies to look at the expression of transient markers in other stratifications of OA using murine models. Surgically induced DMM model is widely used for target validation studies or evaluation of the pathophysiological roles of many molecules in OA. Following DMM, medial displacement of the medial meniscus in a mouse knee joint provides a smaller area to transmit the weight-bearing forces and leads to an increased local mechanical stress (20). Whereas, in a cyclic AC tibial compression model, the non-invasive dynamic mechanical loading applied to the mouse tibia through the knee and ankle joints, modifies AC structure locally through a mechanoadaptive homeostatic response contributing to OA development (14). Hypertrophic chondrocytes in the calcified cartilage and GP of the healthy joints express Col10a1 (14, 19). The calcified cartilage acts to protect the uncalcified AC through maintaining its ECM in an unmineralised state and the stability of the AC. However, hypertrophic differentiation of these chondrocytes contributes to AC matrix degradation, calcification and vascular invasion resulting in the demise of the AC (21). Consistent with this, the expression of Col10a1, as examined using immunohistochemistry, has been observed throughout the AC in the joints of our both DMM and loaded mice. Further, the higher expression level of another marker of chondrocyte hypertrophy; MMP13 has been detected in superficial and uncalcified chondrocytes in the AC of DMM and loaded mice compared to their non-operated and non-loaded left knee joints. Indeed, cartilage degradation observed in OA has been attributed to an elevated production of proteolytic enzymes among which MMP13 has a major role (22, 23). Studies using transgenic mice deficient in catabolic transcription factors that induce hypertrophic differentiation revealed that animals were protected against surgically and chemically induced OA further highlighting the role of transient chondrocytes in AC degradation (24, 25).

The results of the present study indicate that in these murine OA models, there is a significantly enlarged zone of GP proliferative and hypertrophic chondrocytes, compared with the chondrocyte zones in the GP of control tibiae of both models. Similarly, a significantly increased cumulative GP width was observed in these models. Consistent with a previous study by Lamuedra et al., this was associated with aberrant widespread expression of Col10a1 and MMP13 in the GP of DMM (10) and loaded tibiae of C56BL/6 mice, compared to the GP of non-operated and non-loaded control knees. However, contradictory to our findings, Lamuedra et al., found that DMM in 10-week old female mice had no effect on GP thickness, and rather on the columnar structure of the GP (10). Longitudinal bone growth is determined by the modifying number of chondrocytes in the proliferative zone of GP, rate of their proliferation, the extent of chondrocyte hypertrophy and controlled synthesis and degradation of ECM throughout the GP (26). Altered growth rate and mechanical modulation of GP function appear to result from complex interactions of changes in the states of these chondrocytes, as does the rate of GP closure due to the formation of bone bridges forming and spanning the width of the GP. Therefore, the differences between our results and those seen by Lamuedra et al., could be due to the different ages examined, or due to the different sexes of the animals as it is known that DMM in female mice results in less severe articular cartilage damage compared to male mice (27).

Our report of aberrant GP chondrocyte dynamics in these DMM and loaded C57BL/6 mice is further strengthened by our data acquired using a 3D quantification method of bone bridging across the tibial epiphysis (16). Here we show significantly enriched spatial localisation of GP bone bridging clustering and number in the tibial medial compared to the lateral compartments of DMM mice in comparison to non-operated mice. We reveal focal clusters of higher density bridges in different anatomical regions of the tibiae, which was correlated with increased SCB thickness. We postulate that the formation of these bridges may be accelerated by local factors like instigating altered mechanoadaptive response and that these spatial variations in GP bridging may disclose the anatomical vulnerability to OA. These findings are supported by the previous studies suggesting associations between local mechanical stress caused by medial displacement of the medial meniscus (28) or cyclic tibial AC compression (14) and GP function in C57BL/6 mice. They are further supported by our previous work in which we revealed, by finite element modelling, that GP bridges act to dissipate stresses upon loading to the overlying SCB and thus suggest that this contributes to OA seen in these models (16). Indeed, the GP is subject to a variety of mechanical forces placed upon it, and strain distributions are inhomogeneous through the GP with the hypertrophic zone exhibiting low mechanical stiffness. Therefore, precise interplay between GP bridging and mechanics has yet to be determined, specifically in the context of OA and how this may increase OA vulnerability.

Identification of modified GP dynamics underpinning human OA would allow elucidation into OA predisposition and ultimately enable the development of novel and specific therapeutic interventions. Our recent work in the MRC National Survey of Health and Development (NSHD) found that increased height in childhood was associated, albeit modestly, with lower odds of knee OA at age 53 years, as was adult achieved height (29). Concurrent with this, a recent study from offspring in the Avon Longitudinal Study of Parents and Children (ALSPAC) found height tempo (corresponding to pubertal timing) to be strongly associated with the hip shape models which may be related to hip future risk OA (30). Further, several known OA susceptibility single nucleotide polymorphisms (SNPs) have been associated with hip shape in perimenopausal women in the ALSPAC (31). Most intriguingly, when combined with data from other cohorts, the eight SNPs independently associated with hip shape were intimately associated with the process of endochondral ossification (32). Together these data provide further tantalising evidence that there are associations between GP dynamics during adolescence and OA development.

The limitation of this study is that we only examined the GP at one specific time point for each model and therefore more time points are required to fully understand how these GP bridges temporally affect SCB changes and OA pathology, and whether this can indeed be used as a predictive tool for OA. Further, due to complex anatomy of the femoral growth plate and our 3D bridging method being optimized for the tibia, herein we have focused our analysis on the tibia. With its controllability, the intermittent non-invasive mechanical loading model will allow in future to distinguish between short- and long-term effects of various cyclic loading regimens on SCB and trabecular bone parameters of both tibiae and femora as well as AC integrity, GP dynamics and allow correlation of these to initiation and progression of human OA. Indeed, it is known that in the loading model, the short-term intervention is not sufficient to induce significant changes in subchondral thickness (15) and thus longer intervention times for both the DMM and loading models may also prove useful in pursuit of understanding these relationships.

## Conclusions

Our studies add to the growing body of evidence that aberrant GP dynamics may contribute to OA pathology in both surgical and loading mouse OA models, and that similar GP-related osteoarthritic pathological changes happen in different *in vivo* models of secondary (post-traumatic) OA (20) through inducing direct (DMM) or indirect (mechanical loading) injuries to the joints. We reveal for the first time spatial variation of GP bridging in these OA models which may contribute to anatomical variation in vulnerability of OA development. Our GP bony bridging analysis may signify accelerated cartilage-bone transition in these affected joints, advancing our understanding of GP closure mechanisms and how these contribute to the health of the joint. Further, our work yields more insights into the changes in the micro-mechanical environment of the GP and chondrocytes within the GP. This work extends our knowledge on the contribution of the GP in OA development, which is vital if we are to reduce the burden of this global disease.

## Data Availability Statement

The datasets used and/or analysed during the current study are available from the corresponding author on reasonable request.

## Ethics Statement

The animal study was reviewed and approved by the Roslin Institute Animal Users and Research Ethics Committees.

## Author Contributions

HJS, KM, AET, BJ, and KAS contributed to the study design, analysis and interpretation of the data, drafted, critically reviewed, edited and approved the version of the manuscript for publication. All authors contributed to the article and approved the submitted version.

## Funding

We are grateful to Medical Research Council (to KAS; MR/R022240/1), Tenovus Scotland, the Swedish Research Council (to AET; 2013–455) for funding. The funding sources did not influence on the design of the study, collection, analyses, interpretation of data, writing or submission of the manuscript.

## Conflict of Interest

The research leading to these results has received technical support from 3Dmagination Ltd, Didcot, UK. KM is co-founder and director of 3Dmagination Ltd in Oxfordshire, UK, a company which provides training and consultancy in 3D and 4D imaging.

The remaining authors declare that the research was conducted in the absence of any commercial or financial relationships that could be construed as a potential conflict of interest.

## Publisher’s Note

All claims expressed in this article are solely those of the authors and do not necessarily represent those of their affiliated organizations, or those of the publisher, the editors and the reviewers. Any product that may be evaluated in this article, or claim that may be made by its manufacturer, is not guaranteed or endorsed by the publisher.

## References

[1] Murray CJ Lopez AD. The Global Burden of Disease: A Comprehensive Assessment of Mortality and Disability from Diseases, Injuries and Risk Factors in 1990 and Projected to 2020. Harvard School of Public Health, Boston (1990).

[2] Goldring MB Goldring SR. Osteoarthritis. J Cell Physiol (2007) 213:626–34. doi: 10.1002/jcp.21258 17786965

[3] Altman R Asch E Bloch D Bole G Borenstein D Brandt K. Development of Criteria for the Classification and Reporting of Osteoarthritis: Classification of Osteoarthritis of the Knee. Arthritis Rheum (1986) 29:1039–49. doi: 10.1002/art.1780290816 3741515

[4] Pacifici M Koyama E Shibukawa Y Wu C Tamamura Y Enomoto-Iwamoto M. Cellular and Molecular Mechanisms of Synovial Joint and Articular Cartilage Formation. Ann NY Acad Sci (2006) 1068:74–86. doi: 10.1196/annals.1346.010 16831907 PMC2697570

[5] Staines KA MacRae VE Farquharson C. The Importance of the SIBLING Family of Proteins on Skeletal Mineralisation and Bone Remodelling. J Endocrinol (2012) 214:241–55. doi: 10.1530/JOE-12-0143 22700194

[6] Parfitt AM. Misconceptions (1): Epiphyseal Fusion Causes Cessation of Growth. Bone (2002) 30:337–9. doi: 10.1016/S8756-3282(01)00668-8 11856639

[7] Glatt V Canalis E Stadmeyer L Bouxsein ML. Age-Related Changes in Trabecular Architecture Differ in Female and Male C57BL/6J Mice. J Bone Miner Res (2007) 22:1197–207. doi: 10.1359/jbmr.070507 17488199

[8] Staines KA Madi K Mirczuk SM Parker S Burleigh A Poulet B. Endochondral Growth Defect and Deployment of Transient Chondrocyte Behaviors Underlie Osteoarthritis Onset in a Natural Murine Model. Arthritis Rheumatol (2016) 68:880–91. doi: 10.1002/art.39508 PMC4832379 26605758

[9] Madi K Staines KA Bay BK Javaheri B Geng H Bodey AJ. In Situ Characterization of Nanoscale Strains in Loaded Whole Joints *via* Synchrotron X-Ray Tomography. Nat BioMed Eng (2019) 4:343–54. doi: 10.1038/s41551-019-0477-1 PMC7101244 31768001

[10] Lamuedra A Gratal P Calatrava L Ruiz-Perez VL Largo R Harrero-Beaumont G. Disorganization of Chondrocyte Columns in the Growth Plate Does Not Aggravate Experimental Osteoarthritis in Mice. Sci Rep (2020) 10:10745. doi: 10.1038/s41598-020-67518-0 32612184 PMC7329885

[11] Glasson SS Blanchet TJ Morris EA. The Surgical Destabilization of the Medial Meniscus (DMM) Model of Osteoarthritis in the 129/SvEv Mouse. Osteoarthr Cart (2007) 15:1061–9. doi: 10.1016/j.joca.2007.03.006 17470400

[12] Staines KA Ikpegbu E Törnqvist AE Dillon S Javaheri B Amin AK. Conditional Deletion of E11/podoplanin in Bone Protects Against Load-Induced Osteoarthritis. BMC Musculoskelet Disord (2019) 20(1):1–11. doi: 10.1186/s12891-019-2731-9 31351471 PMC6661085

[13] Samvelyan HJ Mathers JC Skerry TM. Feeding Intervention Potentiates the Effect of Mechanical Loading to Induce New Bone Formation in Mice. FASEB J (2021) 35:e21792.1–14. doi: 10.1096/fj.202100334RR PMC12316085 34516688

[14] Poulet B Hamilton RW Shefelbine S Pitsillides AA. Characterizing a Novel and Adjustable Noninvasive Murine Joint Loading Model. Arthritis Rheum (2011) 63:137–47. doi: 10.1002/art.27765 20882669

[15] Poulet B de Souza R Kent AV Saxon L Barker O Wilson A. Intermittent Applied Mechanical Loading Induces Subchondral Bone Thickening That May Be Intensified Locally by Contiguous Articular Cartilage Lesions. Osteoarthr Cartil (2015) 23(6):940–8. doi: 10.1016/j.joca.2015.01.012 PMC4459965 25655679

[16] Staines KA Madi K Javaheri B Lee PD Pitsillides AA. A Computed Microtomography Method for Understanding Epiphyseal Growth Plate Fusion. Front Mater (2018) 4:48. doi: 10.3389/fmats.2017.00048 29417047 PMC5798587

[17] Hunziker EB Schenk RK Cruz-Orive LM. Quantitation of Chondrocyte Performance in Growth Plate Cartilage During Longitudinal Bone Growth. J Bone Joint Surg (1987) 69(2):162–73. doi: 10.2106/00004623-198769020-00002 3543020

[18] Chambers MG Kuffner T Cowan SK Cheah KSE Mason RM. Expression of Collagen and Aggrecan Genes in Normal and Osteoarthritic Murine Knee Joints. Osteoarthr Cartil (2002) 10:51–61. doi: 10.1053/joca.2001.0481 11795983

[19] Poulet B Westerhof TAT Hamilton RW Shefelbine SJ Pitsillides AA. Spontaneous Osteoarthritis in Str/ort Mice Is Unlikely Due to Greater Vulnerability to Mechanical Trauma. Osteoarthr Cartil (2013) 21:756–63. doi: 10.1016/j.joca.2013.02.652 23467034

[20] Samvelyan HJ Hughes D Stevens C Staines KA. Models of Osteoarthritis: Relevance and New Insights. Calcif Tissue Int (2020) 109(3):243–56. doi: 10.1007/s00223-020-00670-x PMC8403120 32062692

[21] Ea HK Nguyen C Bazin D Bianchi A Guicheux J Reboul P. Articular Cartilage Calcification in Osteoarthritis: Insights Into Crystal-Induced Stress. Arthritis Rheum (2011) 63:10–8. doi: 10.1002/art.27761 20862682

[22] Ortega N Behonick DJ Werb Z. Matrix Remodeling During Endochondral Ossification. Trends Cell Biol (2004) 14:86–93. doi: 10.1016/j.tcb.2003.12.003 15102440 PMC2779708

[23] Stickens D Behonick DJ Ortega N Heyer B Hartenstein B Yu Y. Altered Endochondral Bone Development in Matrix Metalloproteinase 13-Deficient Mice. Development (2004) 131:5883–95. doi: 10.1242/dev.01461 PMC2771178 15539485

[24] Saito T Fukai A Mabuchi A Ikeda T Yano F Ohba S. Transcriptional Regulation of Endochondral Ossification by HIF-2α During Skeletal Growth and Osteoarthritis Development. Nat Med (2010) 16:678–86. doi: 10.1038/nm.2146 20495570

[25] Yang S Kim J Ryu JH Oh H Chun CH Kim BJ. Hypoxia-Inducible Factor-2α Is a Catabolic Regulator of Osteoarthritic Cartilage Destruction. Nat Med (2010) 16:687–93. doi: 10.1038/nm.2153 20495569

[26] Breur GJ Lapierre MD Kazmierczak K Stechuchak KM McCabe GP. The Domain of Hypertrophic Chondrocytes in Growth Plates Growing at Different Rates. Calcif Tissue Int (1997) 61:418–25. doi: 10.1007/s002239900358 9351885

[27] Hwang HS Park IY Hong JI Kim JR Kim HA. Comparison of Joint Degeneration and Pain in Male and Female Mice in DMM Model of Osteoarthritis. Osteoarthr Cartil (2021) 29(5):728–38. doi: 10.1016/j.joca.2021.02.007 33609695

[28] Huang H Skelly JD Ayers DC Song J. Age-Dependent Changes in the Articular Cartilage and Subchondral Bone of C57BL/6 Mice After Surgical Destabilization of Medial Meniscus. Sci Rep (2017) 7:1–9. doi: 10.1038/srep42294 28181577 PMC5299455

[29] Staines KA Hardy RJ Samvelyan HJ Ward KA Cooper R. Life Course Longitudinal Growth and Risk of Knee Osteoarthritis at Age 53 Years: Evidence From the 1946 British Birth Cohort Study. Osteoarthr Cartil (2020) 29(3):335–40. doi: 10.1101/2020.08.18.20177485 PMC7955286 33383179

[30] Frysz M Gregory JS Aspden RM Paternoster L Tobias JH. The Effect of Pubertal Timing, as Reflected by Height Tempo, on Proximal Femur Shape: Findings From a Population-Based Study in Adolescents. Bone (2020) 131:115179 1–8. doi: 10.1016/j.bone.2019.115179 PMC6961111 31794847

[31] Baird DA Paternoster L Gregory JS Faber BG Saunders FR Giuraniuc CV. Investigation of the Relationship Between Susceptibility Loci for Hip Osteoarthritis and Dual X-Ray Absorptiometry–Derived Hip Shape in a Population-Based Cohort of Perimenopausal Women. Arthritis Rheumatol (2018) 70:1984–93. doi: 10.1002/art.40584 29882636

[32] Baird DA Evans DS Kamanu FK Gregory JS Saunders FR Giuraniuc CV. Identification of Novel Loci Associated With Hip Shape: A Meta-Analysis of Genomewide Association Studies. J Bone Miner Res (2019) 34:241–51. doi: 10.1002/jbmr.3605 PMC6375741 30320955

